# Challenges and opportunities in country-specific research synthesis: a case study from Cameroon

**DOI:** 10.1186/s13643-017-0552-1

**Published:** 2017-08-08

**Authors:** Lawrence Mbuagbaw, Lynn Cockburn

**Affiliations:** 1 Department of Health Research Methods, Evidence and Impact, McMaster University, Hamilton, ON Canada; 2 Biostatistics Unit, Father Sean O’Sullivan Research Centre, St Joseph’s Healthcare Hamilton, Hamilton, ON L8N 4A6 Canada; 3 Centre for the Development of Best Practices in Health, Yaoundé, Cameroon; 4 Department of Occupational Science & Occupational Therapy, University of Toronto, Toronto, Canada; 5 International Centre for Disability and Rehabilitation, University of Toronto, Toronto, Canada

**Keywords:** Research synthesis, Cameroon, Research output

## Abstract

**Background:**

Research synthesis is an important approach to summarizing a body of literature. Usually, the goal is to determine the effectiveness of an intervention, to determine the strength of association between two factors, to determine the prevalence of a condition, or to scope the literature. Research synthesis methods can also be used to appraise the quantity and quality of research output from institutions or countries. In the latter case, standard quantitative systematic review methodologies would not apply and investigators must borrow strategies from qualitative syntheses and bibliometric analyses to develop a complete and meaningful appraisal of the literature from a given country.

**Methods:**

In this paper, we use the example of Cameroon to highlight some of the challenges and opportunities of appraising a body of country-specific literature. A comprehensive and exhaustive search of the literature was conducted to identify health-related literature from Cameroon published from 2005 to 2014. Titles were screened in duplicate.

**Results:**

A total of 8624 studies were retrieved of which 721 were retained. The main challenges were making a choice of synthesis approach; selecting the right databases, data storage and management; and sustaining the team. Key opportunities include enhanced networking, a detailed appraisal of funding sources, international collaborations, language of publication, and issues with study design. The product is a comprehensive and informative body of evidence that can be used to inform policy with regards to international collaboration, location of research studies, language of publication, knowledge areas of focus, and gaps.

**Conclusion:**

Knowledge synthesis approaches can be adapted for appraisal of country-specific research and offer opportunities for in-depth appraisal of research output.

## Background

### On research synthesis

Research synthesis refers to the systematic search, collection, analyses, and documentation of research. It exists in several forms, primarily distinguished by their focus, scope, and methods. A number of taxonomies have been used to describe the different forms of research synthesis [1, 2]. In health and medicine, systematic reviews with a highly focused research question are most endorsed. Scoping reviews provide an alternative, and attempt to scope the state of the literature using much broader research questions [3]. Other forms of synthesis include narrative reviews and critical interpretive synthesis. What these research synthesis approaches have in common is the sequential procedure of problem formulation, literature search, data evaluation, analysis, interpretation, and presentation [1]. A sister science—bibliometric analysis—defined as a set of methods to quantitatively analyse scientific and technological literature, may offer complementary information to a research synthesis endeavour [4].

The first part of research synthesis—problem formulation—is often critical in determining what methods will be used [5]. Questions of a comparative nature will require studies that include comparative data, like randomized trials and other comparative observational studies. This question formulation will in turn guide the construction of an appropriate search strategy and inform the choice of tools for evaluation, analysis, and interpretation of findings. Many researchers use the PICOT (participants, intervention, comparator, outcome, timeframe) framework to formulate their research questions, applying all or some of its components as needed and as relevant [5].

On some occasions, researchers might be interested in synthesizing the literature to inform practice, policy, and research for a specific geographic region. In this case, the P would refer to all the people living in that geographic region, as opposed to a specific patient population. Such a systematic scoping review would include only data collected from participants within that geographic region. Understandably, no reasonable quantification or qualification can be given to the intervention (or exposure) and comparison components of this framework. For the purposes of such a piece of work, every possible outcome will be potentially relevant, and a timeframe could be applied such that the data are retrieved up to a certain point.

For this type of synthesis, a bibliometric analysis can be considered, if indeed there was a usable collection of literature earmarked as originating from a specific geographic location. This is not often the case, as electronic databases typically cover international literature. For a low- and middle-income country (LMIC) like Cameroon, strategies to retrieve specific information must be fit to purpose.

### On Cameroon

Cameroon is a central African country with two official languages—English and French. Its health system has evolved from the colonial era to present times in stages involving more loco-regional independence and a focus on communities [6]. Recent health reforms have included the Sector-Wide Approach (SWAps) with more local leadership and ownership of externally funded projects and a reorientation of primary health care in the early nineties [6]. With key targets for reducing mortality and morbidity, improving access to health services and enhancing human resource management, the Ministry of Public Health developed eight broad programs to achieve its goals: disease control, reproductive health, health promotion, drugs and essential consumables and reagents, management, service offering and provision, health sector finance, and institutional development [7]. Despite these goals, health outcomes have not been optimal, and health research has been largely overlooked. Only 0.7% of the national health budget (0.1 of the total national budget) is spent on health research. National health research priorities reflect the vast array of conditions affecting Cameroonians [8].

This paper describes issues related to concerted efforts of an international, interdisciplinary team attempting to conduct a comprehensive literature review of health research on a LMIC and engage in knowledge translation to strengthen health systems and services by answering the question: What is the nature of health research (trends, themes, health systems) conducted on the Cameroonian population during 2005–2014?

### Context of research

As part of a multidisciplinary collaboration between Canadian and Cameroonian researchers, the need for a detailed appraisal of where Cameroon stands in terms of health research output emerged naturally from team discussions. We sought to develop projects that would be mutually beneficial to both parties and that would help to strengthen research ties, but were wary of the risk of duplicating research and embarking on projects that did not reflect national health priorities. As all decent research endeavours begin with a review of the literature, we began formulating strategies to know what the lay of the land was in Cameroon. Our questions included how much health research had been published, on what topics, in what languages, and by whom?

## Methods

### Data collection and search strategy

This study produced a collection of health-related literature from Cameroon published during 2005–2014. A comprehensive search of major and relevant databases available through the University of Toronto library (https://www.library.utoronto.ca/) was conducted in January of 2015. Literature was obtained from the following databases/providers: Biomed Central, Elton B. Stephens Co. (EBSCO), Francis, Journal Storage, Popline, Project Muse, Proquest, PubMed, Social Science Abstracts, Scopus, Web of Science, and OVID databases. The tables of contents of two journals published in Cameroon, the African Journal of Integrated Health and African Health Sciences, were also hand searched.

### Data management and data sharing

Citations were downloaded into the reference management system Zotero (https://www.zotero.org/).

#### Inclusion criteria

Citations in the Zotero database were reviewed for duplication. The titles of all papers and the abstract (if necessary) were then reviewed in duplicate to ensure that articles met the following inclusion criteria:

- Indexed between January 1, 2005, and December 31, 2014
- Primary health-related research focusing on humans living in Cameroon
- Published in English or French
- Mixed methods, qualitative, and quantitative studies (experimental and observational) were eligible
- Articles with analysis of primary or secondary data collected in Cameroon. Studies using secondary data were included if an original analysis was conducted on this data (e.g. articles that conduct analysis on data collected from Demographic and Health Surveys were acceptable for inclusion).
- In multi-country studies, where Cameroon was one of two or more countries, the study was included if information and results regarding Cameroon could be extracted independently from data about other countries.

#### Exclusion criteria

The following types of articles and documents were excluded:

- Publications such as commentaries and letters to the editor that did not involve direct contact with participants
- Animal, plant, and basic pharmaceutical/lab studies
- Secondary studies (i.e. systematic reviews)
- Masters and Doctoral theses

## Results

### Citations retrieved

Our search retrieved 8624 studies of which 2452 were remaining after deduplication. After screening, 721 were retained. These citations are kept in a group Zotero database and can be searched using key words, author names, year of publication, and topic. The database can be found here: https://www.zotero.org/groups/cameroon_health_and_disability_research

**Fig. 1:**
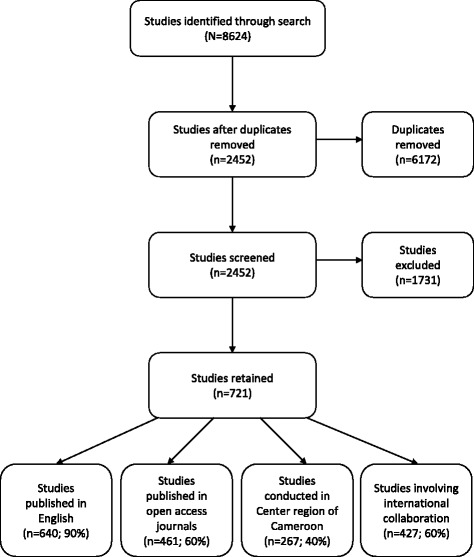
Flow diagram of study selection and key features

A brief overview of this database indicates that close to 60% of the research involves international collaboration (with external co-authors); a relatively stable increase in research output over time; close to 40% conducted in the Centre region of Cameroon (one of ten regions) and about 60% published in open access journals. Almost 90% of the research was published in English. France, USA, and South Africa were the top three international collaborators. As a work in progress, these highlights can be used to inform research planning and policy. Full details of this bibliometric analysis will be reported elsewhere. The study selection and key features of this database are outlined in Fig. 1.

## Challenges

### Determining what synthesis approach to use

We determined early on that we wanted to use a systematic approach to the project. Standard systematic review approaches were insufficient to respond to our needs given that we did not have a focused question that could be meaningfully broken down into all the PICOT elements and that we knew much of the research we wanted to learn about would be excluded in a standard systematic review process. The absence of clear research questions and the pertinence of a broad approach would suggest that a scoping review approach would be useful. However, our need to analyse a specific collection of literature suggested that some aspects of a bibliometric analysis would be useful. We therefore used a combination of both scoping review and bibliometric review methodologies.

### Databases

A good systematic review is expected to include a search of at least two electronic databases [9]. Typically, these databases can be selected based on their size, popularity and content. The US National Medical Library (MEDLINE) covers a large part of the medical literature but includes mostly North American literature. The Excerpta Medica database (EMBASE) is more Europe-centric. The Cumulative Index to Nursing and Allied Health Literature (CINAHL) is a good source for nursing and paramedical articles. Latin American and Caribbean Health Sciences Literature (LILACS) covers the Latin Americas and Caribbean islands. Even though these databases would also index literature from Africa, a large portion of African (and Cameroonian) literature would be missed because many African Journals are not indexed on any of these databases. The African Journals Online (AJOL) database seems to cover many African journals, but the search capacities are limited. We also discovered that many Cameroonian journals no longer exist and that Cameroon does not have any electronic databases for health research. We therefore combined a search of electronic databases with local journal websites to obtain the maximum number of studies.

### Storage

Storage of retrieved data presented equally tough choices. Our initial plan was to collect all our data on an excel sheet that could be used for processing. We soon realized for a multidisciplinary team located in several countries to work on an ever-growing data set, we needed a more dynamic and secure platform. We opted for Zotero which is a free and flexible electronic web-based data management tool with a searchable interface (https://www.zotero.org/) that supports group collaboration. We found it to be very useful for cataloguing information from a wide variety of sources, published in different formats. It allows for data to be downloaded in various formats such as text documents or Excel sheets.

### Tagging and classifying

Having built the database, we were faced with the challenge of tagging and classifying the information. Given the broad spectrum of research included in our data base, we had several options. Articles could be tagged by disease specialty and their sub-categories (e.g. surgery, paediatrics, internal medicine, obstetrics and gynaecology). We found that these categories were not mutually exclusive and were uncertain how to classify some papers, such as oncological papers, that could fall in all three categories. More so, papers covering issues other than personal health, like health systems, adherence to care, and quality of life, did not fit adequately under any of these categories. They could also be classified by the population of interest (e.g. adults, children, or women). This approach led to significant overlap in categories and many articles included all three populations of interest. After significant deliberation, we agreed to classify first by the disease, then by the population of interest. Papers that covered multiple conditions were classified under both categories.

We were also interested in exploring the amount of international collaboration in Cameroonian research. Though conceptually challenging to measure, we planned to analyse author affiliations to determine their geographical locations, determine the extent of collaborations, and the countries with which Cameroonian researchers collaborated the most. Articles with a lead author based in Cameroon were considered as Cameroonian-led. Articles with no foreign authors were tagged as “no international collaboration”. Of course, this approach may miss out other forms of collaboration such as funding or methodological support that did not lead to authorship, but would provide a reasonable way of evaluation research collaboration.

### Team building and sustainability of collaboration

Building a team to work on the project involved careful consideration of interests and potential benefits. We hinged on the pre-existing Cameroon-Canada partnership of the Canadian Coalition for Global Health Research (CCGHR), to identify partners who were willing to support efforts to strengthen the Cameroonian health system by documenting current levels of research output.

We proposed to cover the entire body of biomedical and health research from Cameroon, with the possibility of focusing on specific topics of interest. We found researchers interested in disability, human immune-deficiency virus, and research methods. MSc Occupational Therapy students from the University of Toronto, with dedicated time and resources, and under supervision, used the topic as part of their research project. We sent out frequent updates to keep partners abreast of all developments and to provide opportunities for input and feedback. Even though we were unable to secure funding for the project, we succeeded to build upon existing partnerships to complete it.

## Opportunities

### Enhanced networking and collaboration

Despite the challenges, this project led to the development of a strong network of Cameroonian- and Canadian-based researchers. Collaborative efforts led to multiple projects covering various aspects of under-research topics in Cameroon like mental health and disabilities.

### Appraisal of potential funding sources

In order to ensure sustainability of the project, the Canadian and Cameroonian partners actively sought funding to support research staff and students involved in article screening and data extraction. They compiled a list of potential funders that would be useful for this project and others.

### Database appraisal

We identified a number of peculiarities in our database which require further investigation. For example, it is concerning that close to a third of the research conducted on Cameroonians is not readily accessible to them because it is published in “restricted access” journals. In addition, the distribution of research does not reflect regional population or disease burden, neither does the language of publication (90% of publication in English from a predominantly French-speaking country).

### Research output

We have completed and published one narrative synthesis of the research conducted in Cameroon on functioning and disability. In this paper, we highlight the paucity of research on disability and the associated stigma, limited knowledge and awareness, poor quality of care and hindered employment opportunities for people with disabilities [10]. We plan to explore other fields of research including mental health and HIV.

## Discussion

We employed a novel multidisciplinary approach to research synthesis on health research from Cameroon over a 5-year period, using methods from systematic reviews, scoping reviews, and bibliometric analyses to create a searchable database. Despite the challenges described above, we created a comprehensive collection of health literature from Cameroon.

Categorizing the information led to interesting revelations regarding collaboration, distribution, and access to Cameroonian research. As a more detailed appraisal of this body of research is conducted, we are likely to discover many more insights.

We perceive this database as a dynamic resource for researchers interested in having an overview of Cameroonian research. It can be used to explore trends, study designs, regions of the country studied, and collaborative efforts. We plan to include articles that had not been indexed at the time of the search and newly published articles. Hopefully, this work will provide useful resources for students, researchers, and clinicians in Cameroon and encourage more interest in health research.

## Conclusion

There is room for development of the science of knowledge synthesis fit for purpose, especially with regards to output for country-specific or other geographic locations within countries. Adequate collection, storage, and indexing of health research are important for optimal use.

## Abbreviations

AJOL: African Journals Online (AJOL)
CINAHL: Cumulative Index to Nursing and Allied Health Literature
EMBASE: Excerpta Medica database
HIV: Human immunodeficiency virus
LILACS: Latin American and Caribbean Health Sciences Literature
PICOT: Participants, intervention, comparator, outcome, timeframe
SWAp: Sector-Wide Approach
